# Promoting inflammatory lymphangiogenesis by vascular endothelial growth factor-C (VEGF-C) aggravated intestinal inflammation in mice with experimental acute colitis

**DOI:** 10.1590/1414-431X20154738

**Published:** 2016-04-08

**Authors:** X.L. Wang, J. Zhao, L. Qin, M. Qiao

**Affiliations:** Department of Gastroenterology, Institute of Digestive Disease, Tongji Hospital affiliated to Tongji University, Shanghai, China

**Keywords:** Inflammation, Lymphangiogenesis, Microvessel density, Acute colitis, VEGF-C

## Abstract

Angiogenesis and lymphangiogenesis are thought to play a role in the pathogenesis of inflammatory bowel diseases (IBD). However, it is not understood if inflammatory lymphangiogenesis is a pathological consequence or a productive attempt to resolve the inflammation. This study investigated the effect of lymphangiogenesis on intestinal inflammation by overexpressing a lymphangiogenesis factor, vascular endothelial growth factor-C (VEGF-C), in a mouse model of acute colitis. Forty eight-week-old female C57BL/6 mice were treated with recombinant adenovirus overexpressing VEGF-C or with recombinant VEGF-C156S protein. Acute colitis was then established by exposing the mice to 5% dextran sodium sulfate (DSS) for 7 days. Mice were evaluated for disease activity index (DAI), colonic inflammatory changes, colon edema, microvessel density, lymphatic vessel density (LVD), and VEGFR-3mRNA expression in colon tissue. When acute colitis was induced in mice overexpressing VEGF-C, there was a significant increase in colonic epithelial damage, inflammatory edema, microvessel density, and neutrophil infiltration compared to control mice. These mice also exhibited increased lymphatic vessel density (73.0±3.9 *vs* 38.2±1.9, P<0.001) and lymphatic vessel size (1974.6±104.3 *vs* 1639.0±91.5, P<0.001) compared to control mice. Additionally, the expression of VEGFR-3 mRNA was significantly upregulated in VEGF-C156S mice compared to DSS-treated mice after induction of colitis (42.0±1.4 *vs* 3.5±0.4, P<0.001). Stimulation of lymphangiogenesis by VEGF-C during acute colitis promoted inflammatory lymphangiogenesis in the colon and aggravated intestinal inflammation. Inflammatory lymphangiogenesis may have pleiotropic effects at different stages of IBD.

## Introduction

Inflammatory bowel diseases (IBDs) such as Crohn's disease (CD) and ulcerative colitis (UC) are characterized by chronic intestinal inflammation resulting from host-microbial interactions in genetically susceptible individuals (1). Increasing incidence of IBD in the world has spurred efforts to evaluate novel strategies to reduce inflammation, improve quality of life, and induce remission. Although the major goal of treatment used to be control of symptoms, recent strategies focus on mucosal healing and remission via immunomodulation (2,3).

There is increasing evidence to suggest that IBD could be a vascular disease and the pathology of IBD is thought to be associated with the development/enlargement of new blood (angiogenesis) and lymph (lymphangiogenesis) vessels (4 5 6). Angiogenesis has been shown to promote the inflammatory response via induction of cell entry into the mucosa, and to promote bacterial and foreign antigen invasion (7). Vascular endothelial growth factor-A (VEGF-A) is upregulated during active episodes of IBD and this is associated with endothelial proliferation, and vascular leakage (8). Blocking VEGF-A signaling reduces intestinal inflammation in IBD patients (9). Ang-2 has also been shown to mediate inflammatory angiogenesis (10).

There has been a recent focus on lymphatic vessels and their role in controlling tissue edema, leucocyte exit, bacterial clearance and edema absorption (11). Early descriptions of CD and UC described colon lymphatic congestion, remodeling, and expansion in experimental IBD, supporting lymphangitis as a cause and consequence of IBD (12). A recent study reported that CD and UC were both characterized by increased density of lymphatic vessels and lymphangiogenesis (13). Patients with CD and UC consistently exhibited extensive dilation of the lacteals, resulting from lymphatic obstruction and submucosal edema (6,14). However, although the role of lymphangiogenesis has been extensively described in experimental and clinical IBD, the exact molecular mechanisms underlying the lymphatic changes in IBD remain unclear (12).

Current studies on lymphatic vessels in IBD suggest that disturbances in lymphatic function exacerbate IBD. Binding of vascular endothelial growth factors VEGF-C and VEGF-D to the vascular endothelial growth factor receptor-3 (VEGFR-3) was shown to trigger the signaling pathway resulting in lymphangiogenesis (15). Inhibition of the VEGFR-3 signaling pathway exacerbated skin inflammation, while stimulation of VEGFR-3 by VEGF-C156S, a specific VEGFR-3-activator, induced growth of lymphatic vessels and inhibited inflammation in an experimental mouse model (16). Additionally, mice treated with anti-VEGFR-3 antibodies exhibited a significant increase in the number of enlarged lymphatic vessels in the colon submucosa, accompanied by a significant decrease in the severity of inflammation compared to control mice (17). Although these data suggested that lymphangiogenic remodeling may be beneficial at least in early phases of the disease, it was not clear if therapeutic strategies targeting lymphatic vessels would be effective (12,18). In the present study, we investigated the role of lymphatic vessels in acute inflammation of IBD by establishing an acute colitis mouse model overexpressing VEGF-C or VEGF-C156S in the colon tissue. We compared VEGFR-3 expression, lymphatic and blood vessel morphology, colon edema, and infiltration of inflammatory cells during acute inflammation in experimental mice with those of control mice. We aimed to expand our understanding of the role of inflammatory lymphangiogenesis on intestinal inflammation in acute colitis.

## Material and Methods

### Mice

Forty eight-week-old specific pathogen-free (SPF) female C57BL/6 mice were purchased from the Shanghai Laboratory Animal Center, China. The mice were housed under SPF conditions at the Animal Experimental Center in Tongji Hospital, and bred in a conventional SPF facility. All animals were given *ad libitum* access to food, and were given water according to experimental requirements. All animal experiments were conducted in accordance with the Guidelines for the Care and Use of Laboratory Animals of Tongji University. Mice were euthanized by CO_2_ inhalation, followed by cervical dislocation.

### Construction and expression of recombinant adenoviruses encoding the VEGF-C

The adenovirus vector pAD-VEGF-C-IRES-EGFP was constructed by cloning the gene encoding human VEGF-C (GenBank accession NM_005429.2) under the cytomegalovirus promoter in the pAD/CMV/V5-DEST vector. Human embryonic kidney 293 cells were used to produce replication-deficient recombinant adenovirus, which was then concentrated. The titer of recombinant adenovirus (AD-VEGF-C-EGFP) obtained was 1.75×10^11^ plaque-forming units (PFU)/mL. Empty vector AD-EGFP was used as the control and was amplified to a titer of 1×10^10^ PFU/mL. Real time quantitative-PCR (qPCR) was used to determine the expression of AD-VEGF-C-EGFP *in vitro*. The primers for VEGF-C were 5′-GTGCATGAACACCAGCACGAG-3′ (forward), 5′-TCCAGCATCCGAGGAAAACA-3′ (reverse). The relative amount of specific mRNA was normalized to human GAPDH using the following GAPDH primers: 5′-GGGTGTGAACCATGAGAAGTATG-3′ (forward), 5′-GATGGCATGGACTGTGGTCAT-3′ (reverse). Fluorescence microscopy was used to evaluate virus localization in the intestines of three healthy mice.

### Experimental design

Acute distant colitis was induced in female C57BL/6 mice (n=10 per group) by providing them with 200 mL of a solution of filtered water containing 5% dextran sodium sulfate (DSS; MW 36,000-50,000; MP Biomedical, USA) *ad libitum* for 7 days, as previously described (19). The DSS solution was changed every other day. Mouse weight, stool form, occult blood test results and water consumption (mL) were recorded daily. On day 7, mice were sacrificed by CO_2_ inhalation, followed by cervical dislocation. Colonic tissue samples were harvested by cutting 1.0 to 1.5 cm long colonic fragments after making note of whether the samples were from the proximal, middle, or distal regions.

Mice in the VEGF-C group were injected in the tail vein with AD-VEGF-C-EGFP (1×10^8^ PFU), while mice in the DSS group were injected with AD-EGFP 2 days prior to the administration of DSS. Control mice received drinking water with no DSS added. Virus localization was evaluated by fluorescence microscopy in frozen sections, which were prepared from 3 healthy mice after 8 days.

The effect of AD-VEGF-C was confirmed using recombinant human VEGF-C156S protein, which is a selective agonist of VEGFR-3 where the characteristically spaced cysteine residues in the VEGF homology domain (Cys156) are replaced with serine residues. VEGF-C156S has been shown to induce lymphangiogenesis but not angiogenesis. Mice (n=5) received a daily intraperitoneal injection (250 µL) of recombinant VEGF-C156S (1 µg/g) diluted in sterile phosphate-buffered saline (PBS) containing 0.1% human serum albumin. Control mice (n=5) received a daily intraperitoneal injection of rat IgG (1 µg/g) in 250 µL sterile PBS solution. The specimens were fixed in 10% formalin for histological analysis by hematoxylin/eosin (H&E) and immunohistochemical staining. All experiments were repeated three times.

### Assessment of colitis severity

The disease activity index (DAI) was evaluated daily during the duration of the DSS treatment by an unbiased observer who had no information about the experiment. DAI was assessed using previously published scoring systems (20,21). DAI was determined using the combined score of weight loss compared to initial weight, stool consistency, and bleeding. Scores were defined as: W) weight loss: 0 (<1%), 1 (1-5%), 2 (5-10%), 3 (10-15%), and 4 (>15%); S) stool consistency: 0 (normal), 2 (loose stools), and 4 (diarrhea); B) bleeding: 0 (no blood), 1 (hemoccult positive), 2 (hemoccult positive and visual pellet bleeding), and 4 (gross bleeding, blood around anus). Stool consistency was assessed using a pair of forceps and pressing down on the feces. Presence of blood in the feces was evaluated by noting the color of the feces (i.e., black stool versus light brown stool) and further validated using the Hemoccult test kit (Nanjing Jiancheng Technology Co., Ltd., China). The final macroscopic score for each animal was the sum of each individual score.

To evaluate histological damage of colitis severity, 0.5 cm fragments from the distant section of the colon (1.0 cm) were submerged in 10% buffered formalin solution. Paraffin-embedded sections (5-µm thick) were stained with H&E using standard procedures. The colonic tissue sections were independently scored by 2 pathologists using a previously published system (20). The scoring was performed as follows: crypt architecture (normal, 0; severe crypt distortion with loss of entire crypt, 3); degree of inflammatory cell infiltration (normal, 0; dense inflammatory infiltration, 3); muscle thickening (base of crypt sits on the muscularis mucosa, 0; marked muscle thickening present, 3); goblet cell depletion (absent, 0; present, 1); crypt abscess (absent, 0; present, 1). The histological damage score was the sum of each individual score. It should be noted that crypt abscess and microscopic ulceration are rare in the DSS-induced murine colitis model.

### Immunohistochemical and histomorphometric analysis

For immunohistochemical staining, sections of the distant colon were cut (4-µm thick) from each study block. Three colon rings were obtained from each colon segment. Antigen retrieval was performed by heating the slides in a microwave oven in a solution of 0.01 mM sodium citrate (pH 6.0). Sections were treated for 25 min at room temperature with 0.3% H_2_O_2_ to block the endogenous peroxidase. Samples were blocked with 3% bovine serum albumin at room temperature, and then incubated at 4°C overnight in a humidity tray with a 1:50 dilution of goat anti-mouse CD31/PECAM-1 polyclonal antibody (GB13063, Wuhan Goodbiotechnology Co. Ltd. China), or a 1:200 dilution of rabbit anti-mouse LYVE-1 polyclonal antibody (ab14917, Abcam, USA). Slides were rinsed thrice for 2 min in 0.1 mM phosphate-buffered saline (PBS), and incubated for 30 min at room temperature with goat anti-rabbit/rabbit anti-goat horseradish peroxidase (HRP, Envision, Dako, USA). Color was developed with 3′3-diaminobenzidine. The primary antibody was replaced with normal goat or rabbit IgG in the negative controls. LYVE-1-positive structures with lumina were defined as lymphatic vessels (17,22).

Vessels positive for LYVE-1 were inspected by light microscopy at 10× magnification. Five areas of tissue with the highest density of lymphatic vessels were selected ("hot areas"). Lymphatic vessel density (LVD) was assessed by counting all stained vessels in these regions and the mean number of the total counted vessels determined as LVD. CD31-positive stained vessels with lumina were defined as microvessels. The mean microvessel density (MVD) was assessed in the same manner as LVD. The thickness of the colon submucosa (inflammatory edema) was evaluated by measuring the width of the colon submucosa area, which encompasses the lamina propria at the basis of the epithelial crypts, the muscularis mucosa, submucosa, and muscle layers (17). Histomorphometric analyses were performed using NIS Elements 4.0 Microscope Imaging Software (Nikon, USA). Scoring and counting were performed independently by two investigators who had no clinical information about the animals.

### Immunofluorescence analysis

Antigen retrieval for immunofluorescence experiments was performed on paraffin-embedded sections as described above. The samples were then incubated overnight at room temperature with a 1: 100 dilution of CD45 antibody (Abcam, UK), a 1:50 dilution of CD3, myeloperoxidase (MPO), CD11C antibodies (Abcam), anti-mouse LYVE-1 (as described above), or a 1:50 dilution of goat anti-mouse CD31 polyclonal antibody (GB13063, Wuhan Goodbiotechnology Co., Ltd.). After three washes with PBS, the slides were incubated for 60 min at room temperature with goat anti-rabbit or goat anti-mouse 488-Alex labeled secondary antibodies (Wuhan Goodbiotechnology Co., Ltd). The nuclei were counterstained with DAPI. The samples were examined under an epifluorescence microscope (Nikon, Japan) and digital pictures were captured. To quantify the positive staining in terms of cell numbers per millimeter of colon tissue, images of 3 or 4 individual fields of view were acquired per sample.

### Quantitative real-time RT-PCR

Total RNA was extracted from mouse colon tissue using Trizol reagent (Invitrogen Life Technologies, USA) according to the manufacturer's instructions. Total RNA (2 µg) was used as a template for first-strand DNA synthesis using the Revert Aid First Strand cDNA Synthesis Kit for reverse transcription (Invitrogen). Real-time RT-PCR was used to determine the expression of mouse VEGFR-3 mRNA with the Toyobo Thunderbird SYBR qPCR Mix (Lifescience, Toyobo Bio-Technology, Co. Ltd., Japan). The reaction mix contained 12.5 µL of Real-Time PCR Master Mix, 0.5 µL 2×SYBR Premix Ex Taq (Toyobo Biotech Co. Ltd.), 2.5 µL of primer mix, 2.0 µL of cDNA and 8.0 µL ddH_2_O. The primers for VEGFR-3 (NM_053652, 95 bp) were 5′-TGAAAGACGGCACACGAATG-3′ (forward), and 5′-CCTCGCTTTAGGGTCTCCAG-3′ (reverse). The relative amount of specific mRNA was normalized to human β-actin (NM_031144, 110 bp) using the following primers: 5′-CGTTGACATCCGTAAAGACCTC-3′ (forward), 5′-TAGGAGCCAGGGCAGTAATCT-3′ (reverse). All the primers were designed by Invitrogen Biotechnology Co., Ltd. All primers spanned an intron to ensure discrimination between cDNA and genomic DNA. PCR cycling conditions were 95°C for 1 min, followed by 40 cycles of 95°C for 15 s, 58°C for 20 s, and 72°C for 20 s. The relative mRNA expression of VEGFR-3 was calculated using the 2–??Ct method and analyzed with the Icycler version 3.1.7050 software (Bio-Rad, USA).

### Western blotting

Total proteins (100 µg) were extracted from colonic mucosa in a lysis buffer containing protease inhibitors (Wuhan Goodbio Co. Ltda) and processed using standard procedures. Protein concentrations were determined using the BCA Protein Assay Kit (Pierce, USA) according to the manufacturer's instructions. Proteins were separated by SDS-PAGE and transferred to polyvinylidene fluoride membranes. Western blot analysis was performed using a 1:1000 dilution of VEGF-C antibody (Santa Cruz Biotechnology, USA), a 1:1000 dilution of VEGFR-2 antibody (CST, USA), or a 1:1000 dilution of VEGFR-3 antibody (Genetex, USA). Loading amounts were normalized using a 1:1000 dilution of anti-actin rabbit polyclonal antibody (Santa Cruz). The density of Western blot bands was measured by densitometry (Alpha EaseFC, Alpha Innotech USA) and normalized to Actin.

### Statistical analysis

Data are reported as mean and standard deviation. Testing of mean differences among groups was performed by analysis of variance (ANOVA). Differences between groups were analyzed by *post hoc* multiple comparisons with the Bonferroni's correction. Mean differences between two groups were compared by two-sample *t*-test. All statistical analyses were done using SPSS statistical software version 22 for Windows (IBM Corp., USA). Figures were generated by GraphPad Prism 6 (GraphPad Software, Inc., USA). A two-tailed P value <0.05 was considered to be significant.

## Results

### Expression of VEGF-C *in vitro* and *in vivo*

Human embryonic kidney 293 cells were transfected with AD-VEGF-C-EGFP, and qPCR was used to detect the expression of VEGF-C *in vitro*. Expression of VEGF-C was significantly upregulated in AD-VEGF-C-EGFP-infected 293 cells compared to AD-EGFP-infected or uninfected cells (P*<*0.001; Supplementary Figure S1A). Fluorescence microscopy was used to localize AD-VEGF-C in the distal and proximal colon tissue of mice injected with AD-VEGF-C-EGFP (Supplementary Figure S1B and C, respectively).

### Overexpression of VEGF-C in the colon mucosa aggravated colonic inflammation during acute experimental colitis in mice

**Figure 1:**
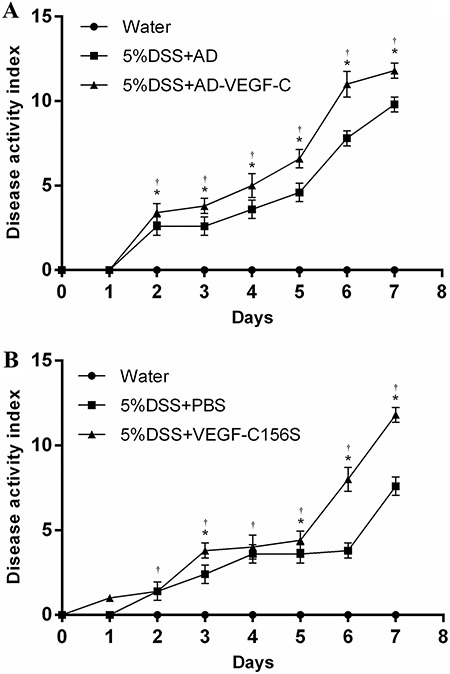
Evaluation of disease activity index (DAI) in AD-VEGF-C-treated mice. Mean DAI scores (±SD) were higher in the AD-VEGF-C-treated mice (*A*) and in recombinant VEGF-C156S-treated mice (*B*), compared to DSS-treated mice. The difference in DAI scores between AD-VEGF-C-treated mice and DSS-treated mice became significant on the second day of observation and continued to differ thereafter. There was a significant difference in DAI scores between recombinant VEGF-C156S-treated mice and PBS-treated mice on days 3, 5, 6 and 7. *P<0.05 between AD-VEGF-C-treated and DSS-treated mice and between VEGF-C156S-treated mice and PBS-treated mice (n=5/group). ^†^P<0.05 compared to water-treated mice (n=5/per group). Statistical analysis was performed by ANOVA and Bonferroni's *post-hoc* test. AD: adenovirus; PBS: phosphate-buffered saline; AD-VEGF-C: adenovirus vascular endothelial growth factor-C; DSS: dextran sodium sulfate.

Experimental colitis was induced in AD-VEGF-C group and the DSS group as described above. DAI scores were assessed daily and averaged for each group. AD-VEGF-C-treated mice had significantly higher DAI scores from the second day of treatment compared to DSS-treated mice (P=0.046 on day 2, P=0.002 on day 3, P=0.003 on day 4, and P<0.001 afterwards) (Figure 1A). VEGF-C156S-treated mice also had significantly higher DAI scores on days 3, 5, 6 and 7 compared to PBS-treated mice (all P≤0.046; Figure 1B).

**Figure 2:**
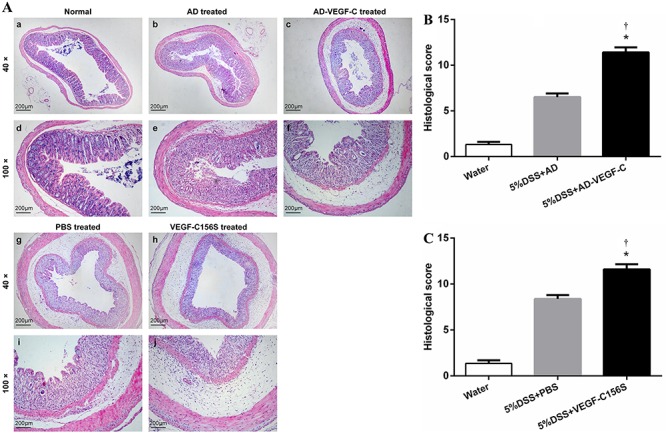
*A*, Assessment of histological scores in the different groups. Histology of normal tissue is shown in panels *a* and *d*, acute colitis was induced with DSS in DSS-treated mice (panels *b* and *e*); in AD-VEGF-C-treated mice (panels *c* and *f*); in PBS-treated mice (panels *g* and *i*); and in VEGF-C156S-treated mice (panels *h* and *j*). DSS-treated mice showed significantly greater histological damage (cellular infiltration, goblet cell depletion, damage to crypt architecture and submucosal edema) (*A: b*, *e*, *g*, *i*) compared to normal mice. VEGF-C-treated mice exposed to DSS showed significantly higher histological scores with more severe histological damage compared to control DSS-treated mice, both in the AD-VEGF-C (*B*, P<0.001) and AD-VEGF-C156S groups (*C*, P<0.001). Data are reported as means±SD. *P<0.05 compared to DSS- or PBS-treated mice (n=5/per group). ^†^P<0.05 compared to water-treated mice (n=5/group). Statistical analysis was performed by ANOVA and Bonferroni's *post-hoc* test. AD: adenovirus; PBS: phosphate-buffered saline; AD-VEGF-C: adenovirus vascular endothelial growth factor-C; DSS: dextran sodium sulfate.

DSS-treated mice showed significantly greater histological damage (cellular infiltration, goblet cell depletion, damage to crypt architecture and submucosal edema) (Figure 2A-b, e, g, i) compared to normal untreated mice (Figure 2A-a, d). AD-VEGF-C-treated mice and VEGF-C156S-treated mice (Figure 2A-c, f, h, j) also showed greater histological damage compared to DSS-treated mice. There was a more severe loss of crypt structure and eroded surface epithelium in the colon of AD-VEGF-C-treated mice compared to DSS-treated mice (11.4±0.5 *vs* 6.5 ±0.4; P<0.001; Figure 2B). VEGF-C156S-treated mice also had higher histological scores, more infiltration of inflammatory cells and more tissue damage compared to PBS-treated mice (11.6±0.5 *vs* 8.4±0.4; P<0.001; Figure 2C).

### Lymphatic and blood vessel remodeling during acute colitis

**Figure 3:**
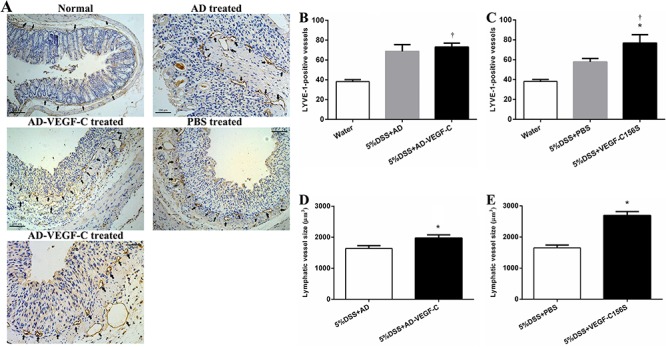
Immunohistochemical results for lymphatic remodeling in acute colitis (*A*). Comparison of lymphatic vessel density (LVD) between AD-VEGF-C treated and control mice (*B*) and between AD-VEGF-C156S-treated and control mice (*C*). AD-VEGF-C did not induce an increase in LVD (P=0.50), whereas VEGF-C156S induced a 1.3-fold increase in LVD compared to PBS-treated mice. Comparisons of lymphatic vessel size between AD-VEGF-C-treated and DSS-treated mice are shown in panel *D* and between AD-VEGF-C156S-treated and PBS-treated mice are shown in panel *E*. Both AD-VEGF-C and VEGF-C156S-treated mice had significantly greater lymphatic vessel size compared to control mice (both P<0.001). Data are reported as means±SD (n=5/group). ^*^P<0.05 compared to DSS-treated group. ^†^P<0.05 compared to water-treated group. Statistical analysis was performed by ANOVA and Bonferroni's *post-hoc* test for lymphatic vessel density and two-sample *t*-test for lymphatic vessel size. AD: adenovirus; PBS: phosphate-buffered saline; AD-VEGF-C: adenovirus vascular endothelial growth factor-C; DSS: dextran sodium sulfate.

Figure 3A shows representative images of immunohistochemical evaluation of lymphatic remodeling during acute colitis. There was no difference in lymphatic vessel density (LVD) between the AD-VEGF-C-treated and DSS-treated mice (73.0±3.9 to 68.6±6.9, P=0.500), whereas AD-VEGF-C+DSS mice had a significantly higher LVD compared to water-treated mice (73.0±3.9 to 38.2±1.9, P<0.001; Figure 3B). However, there was a 1.3-fold increase in LVD in the VEGF-C156S-treated mice compared to PBS-treated mice (76.8±8.4 to 57.8±3.5, P<0.001; Figure 3C).

The lymphatic vessels induced by VEGF-C were enlarged and tortuous, and were mainly located in the mucosa and the lamina propria. The AD-VEGF-C-treated mice had a significantly larger lymphatic vessel size compared to DSS-treated mice (1974.6±104.3 to 1639.0±91.5, P<0.001; Figure 3D). Similarly, the VEGF-C156S-treated mice had a significantly larger lymphatic vessel size as compared to the PBS-treated mice (2690.4±125.8 to 1650.8±93.2, P<0.001; Figure 3E).

**Figure 4:**
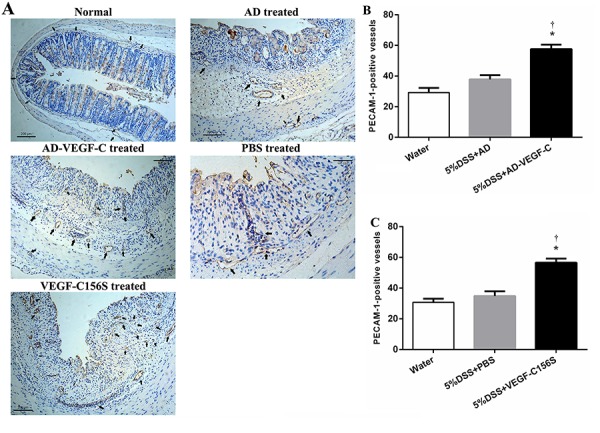
Immunohistochemical results for blood vessel remodeling in acute colitis (*A*). Comparison of microvessel density between AD-VEGF-C treated and DSS-treated mice (*B*) and between AD-VEGF-C156S-treated and PBS-treated mice (*C*). Both AD-VEGF-C and VEGF-C156S induced a significant increase in microvessel density compared to DSS-treated mice (both P<0.001). Data are reported as means±SD (n=5/per group). *P<0.05 compared to DSS- or PBS-treated mice. ^†^P<0.05 compared to water-treated mice. Statistical analysis was performed by ANOVA and Bonferroni's *post hoc* test. AD-VEGF-C: adenovirus vascular endothelial growth factor-C; DSS: dextran sodium sulfate. PECAM 1: polyclonal antibody.

Figure 4A shows representative images of immunohistochemical evaluation of blood vessel remodeling in the acute colitis. There was a 1.5-fold and a 1.6-fold increase in MVD in the AD-VEGF-C-treated mice and VEGF-C156S-treated mice compared to the DSS-treated mice (57.6±2.9 to 37.8±2.9, and 56.6±2.6 to 34.8±3.1, respectively; both P<0.001; Figure 4B and C).

### High expression of VEGFR-3 during acute colon inflammation

**Figure 5:**
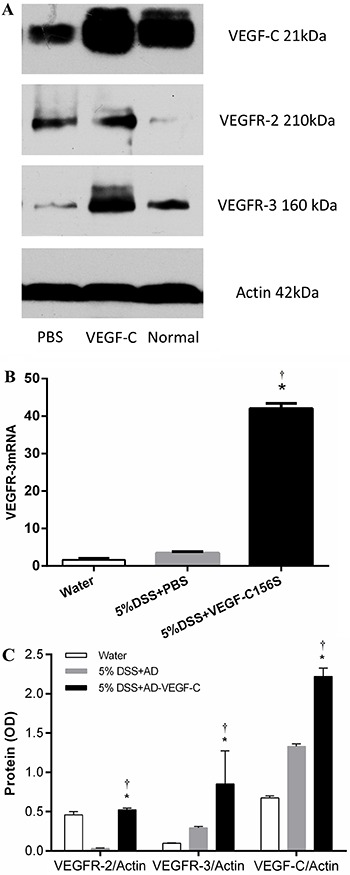
*A*, Western blot of VEGF-C, VEGFR-2 and VEGFR-3 in the different experimental groups. *B*, Comparison of VEGFR-3 mRNA expression detected by quantitative real-time RT-PCR. VEGFR-3 mRNA levels were significantly upregulated in VEGF-C156S-treated mice compared to DSS-treated mice (P<0.001) as well as water-treated mice (P<0.001). *C*, Quantitative comparison of Western blot of VEGFR-2, VEGFR-3 and VEGF-C between experimental groups. VEGF-C expression was upregulated in AD-VEGF-C-treated mice compared to DSS-treated mice (P<0.001). DSS-treated mice had higher levels of VEGF-C expression compared to water-treated mice (P<0.001). VEGFR-3 expression in colonic mucosa was higher after VEGF-C treatment compared to mice treated with water (P=0.001) or DSS (P=0.01). Data are reported as means±SD. *P<0.05 compared to 5% DSS-treated mice. ^†^P<0.05 compared to water-treated mice (n=5/group). Statistical analysis was performed by ANOVA and Bonferroni's *post-hoc* test. AD-VEGF-C: adenovirus vascular endothelial growth factor-C; DSS: dextran sodium sulfate.

We investigated whether high VEGF-C expression affected the expression of its receptor, VEGFR-3, on lymphatic endothelium of mice with acute colitis. We used VEGF-C156S-treated mice and determined the expression of VEGFR-3 mRNA using real-time RT-PCR. Although VEGFR-3 mRNA levels were slightly up-regulated in 5% of the DSS-treated mice compared to normal control mice (P=0.014), they were significantly and dramatically upregulated in VEGF-C156S-treated mice compared to DSS-treated mice (42.0±1.4 *vs* 3.5±0.4, P<0.001) as well as control mice (42.0±1.4 *vs* 1.6±0.4, P<0.001; Figure 5B).

Western blotting was used to further investigate the effect of VEGF-C on VEGFR-3 protein expression. DSS-treated mice had higher levels of VEGF-C expression compared to control mice (P<0.001). In addition, VEGF-C expression was upregulated in AD-VEGF-C-treated mice compared to DSS-treated mice (P<0.001). VEGFR-3 expression in colonic mucosa was higher after VEGF-C treatment compared to mice treated with water (0.85±0.42 *vs* 0.10±0.01, P=0.001) or DSS (0.85±0.42 *vs* 0.29±0.02, P=0.01; Figure 5C).

### Inflammatory cells infiltration in acute experimental colitis

Recruitment of inflammatory cells was investigated in the two models of acute colitis after VEGF-C overexpression. Supplementary Figure S2A shows representative fluorescence micrographs of CD11c-positive dendritic cells after DSS-induced acute colitis. AD-VEGF-C-treated and VEGF-C156S-treated mice both had significantly higher numbers of CD11c-positive dendritic cells compared to DSS-treated mice (32.0±1.9 *vs* 18.8±2.2; P=0.004, and 32.6±2.0 *vs* 22.4±1.7; P=0.002, respectively) (Supplementary Figure S2B and C). Supplementary Figure S3A shows representative fluorescence micrographs of MPO-positive neutrophils in mice with DSS-induced acute colitis. There was no significant difference in the number of MPO-positive neutrophils in the colonic mucosa between the AD-VEGF-C-treated and DSS-treated mice (P=0.897; n=5) (Supplementary Figure S3B). However, the VEGF-C156S-treated mice had a significantly higher number of MPO-positive neutrophils in the colonic mucosa compared to DSS-treated mice (30.0±1.6 *vs* 23.2±2.3, P=0.018; Supplementary Figure S3C).

### Increase in edema formation during acute colitis after activation of lymphatic vessels

The effect of lymphatic vessel activation on acute colitis was investigated and AD-VEGF-C-treated mice had significant thicker colons compared to mice treated only with DSS (397.4±2.3 *vs* 284.2±11.7; P<0.001). Similarly, VEGF-C156S-treated mice had significantly thicker colons compared to PBS control mice (484.4±10.2 *vs* 291.2±50.2; P<0.001; Supplementary Figure S4A and B, respectively).

## Discussion

In this study, we showed that when acute colitis was induced in mice overexpressing VEGF-C, there was a significant increase in colonic epithelial damage, inflammatory edema and neutrophil infiltration compared to control mice. These mice also exhibited increased LVD, and enlarged lymphatic vessel size compared to control mice. Additionally, the expression of VEGFR-3 was significantly upregulated in VEGF-C156S mice after induction of colitis compared to control mice. Our data suggested that VEGF-C/VEGFR-3 signaling during acute colitis promoted inflammatory lymphangiogenesis in the colon and aggravated intestinal inflammation.

Lymphangiogenesis has been shown to be a characteristic feature of acute, as well as chronic, inflammatory diseases (18). Although regulation of lymphangiogenesis has been demonstrated in experimental models of chronic inflammatory diseases such as chronic skin inflammation (16), chronic inflammatory arthritis (23), and corneal inflammation (24), the functional role of the lymphatic vasculature in acute inflammation remains unclear. It is also not understood if inflammatory lymphangiogenesis represents the pathology of inflammation, or a productive attempt to resolve the inflammation.

Our data showing that VEGF-C-induced lymphangiogenesis promoted intestinal inflammation were consistent with previous studies demonstrating lymphangiogenesis in inflammatory diseases such as psoriasis and chronic airway inflammation (25,26). Our data also agreed with studies showing that lymphangiogenesis was frequently associated with transplant rejection of kidney or cornea (27,28). However, our data were in contrast with other studies demonstrating that 1) systemic delivery of VEGF-C provided significant protection against inflammation in a DSS model of acute and chronic colitis (29); 2) VEGFR-3 blockade significantly reduced lymphatic vessel density, while significantly increasing inflammatory edema formation and inhibiting disease resolution (29); 3) transgenic delivery of VEGF-C/-D significantly induced lymphangiogenesis (30), and limited acute skin inflammation via enhanced lymphatic drainage and reduction of edema formation (31).

We suggest that the discrepancy between our data and these studies can be explained by the fact that the microvascular and lymphatic endothelium in the gut have complementary functions (6). C57BL/6 mice exposed to DSS were shown to develop acute colitis with a significant increase in blood vessel density in the mucosa and submucosa layers during the acute phase of inflammation (10). In the present study, VEGF-C-treated mice also had a significant increase in blood vessel density and dilation of blood vessels compared to control mice, suggesting that VEGF-C specifically stimulated the VEGFR-3 signaling pathway to induce angiogenesis during acute colitis. This could have important consequences since the new blood vessels formed during acute colitis have been shown to be leaky and more responsive to stimulation by growth factors, which promote recruitment of additional inflammatory cells to the site of inflammation (32,33).

Acute colitis was previously shown to be characterized by an overall higher number of lymphatic vessels compared to blood vessels (10), and the greater lymphatic expansion was thought to compensate for leaky blood vessels (13). In the present study, although VEGF-C-treated mice had a higher LVD compared to DSS-treated mice, the intestinal inflammation in these mice was not resolved, possibly because the microvascular endothelium overloaded the inflammatory burden, causing a failure of the lymphatic vasculature to carry this burden away from affected gut segments. At the subclinical phase, these results suggest that, although the increasing lymphatics can limit inflammation, it is a challenge to resolve intestinal inflammation when it is overburdened. Failure of increased LVD to resolve colon inflammatory edema and infiltration of neutrophils could also be attributed to high interstitial pressure and lymphatic endothelium dysfunction, which could result in failure of lymphatic vessel drainage (6). Since lymphangiogenesis is thought to represent a protective/adaptive response, we suggest that promoting lymphangiogenesis at a very early stage of acute colitis, before the onset of events leading to severe edema and infiltration, would have maximal therapeutic value.

A number of cellular mediators including macrophages, neutrophils and dendritic cells have been shown to play a role in regulating inflammatory lymphangiogenesis via secretion of VEGFs (34). Our study demonstrated significantly increased numbers of neutrophils in VEGF-C-treated mice compared to DSS-treated mice. The complex interplay between angiogenesis and lymphangiogenesis is underscored by the finding that, although neutrophil infiltration is triggered by angiogenesis (35), neutrophils modulate lymphangiogenesis via VEGF-A and VEGF-D in different inflammatory models (36 37 38). Interestingly, although the exact mechanism was not clear, B cell-induced lymphangiogenesis was previously shown to be associated with increased mobilization of dendritic cells (39). Lymphatic vessels have been shown to play a role in transport of dendritic cells to draining lymph nodes (31) and inflamed lymphatic endothelium was shown to suppress dendritic cell maturation and function (40). Our study showed that VEGF-C treated mice had higher numbers of CD11c+ dendritic cells compared to DSS-treated mice. Although VEGF-C stimulated the growth of lymphatic vessels during experimental acute colitis, the lack of attenuation of inflammation during lymphangiogenesis, and reduced drainage of dendritic cells during acute inflammation could be a result of defective lymphatic vessel function.

Macrophages and different subsets of T cells have been shown to regulate lymphangiogenesis in lymph nodes during inflammation via secretion of interferon-γ (34). However, we found no evidence of a relationship between intestinal inflammation and the presence of CD3+ T cells, CD45/B220+ B cells, or F4/80 + macrophages. Lymphatic vessels at different sites may have different functions, as evidenced by their different clinical appearance in UC and CD. Lymphangiogenesis could have diverse functional consequences on inflammation since the role of inflammatory lymphangiogenesis may differ based on the inflammatory state (acute or chronic), time frame of its occurrence, as well as site of inflammation (6). It is also possible that, in addition to the VEGF-C/VEGFR-3 signaling pathway, other signaling pathways could play a role in inflammatory lymphangiogenesis. Based on the finding that the balance between pro- and anti-lymphangiogenic factors regulates lymphatic vascular homeostasis (37), it is possible that the immune response in inflammatory diseases such as IBD could be regulated by manipulating the concentrations of these factors.

We believe that the contrast between our data and previous studies, which showed the therapeutic value of VEGF-C for IBD, could be due to the more aggressive DSS colitis model used in this study. The dual effects of VEGF-C make it very important to evaluate its utility as a therapeutic option for IBD. One limitation of the present study was that, due to the lack of a commercially available VEGF-C antibody, which can be used on murine specimens for confocal microscopy, we could not determine the identity of the cells that produced VEGF-C. Further studies are necessary to understand the role and functions of lymphangiogenesis in IBD.

## Supplementary Material


