# Supplementary material for: Promoting inflammatory lymphangiogenesis by vascular endothelial growth factor-C (VEGF-C) aggravated intestinal inflammation in mice with experimental acute colitis
Source: Braz J Med Biol Res. 2016 Apr 8;49(5):e4738. doi: 10.1590/1414-431X20154738 (PMC4830025; doi:10.1590/1414-431X20154738)

Figure S1. *A*, Representative fluorescence micrographs show AD-VEGF-C-EGFP expression in distal colon (*right panel*) and proximal colon (*left panel*). *B*, High expression of recombinant adenovirus AD-VEGF-C in 293 cells detected by real time qPCR. Data are reported as means±SD (n=5/per group). AD-VEGF-C: adenovirus vascular endothelial growth factor-C.

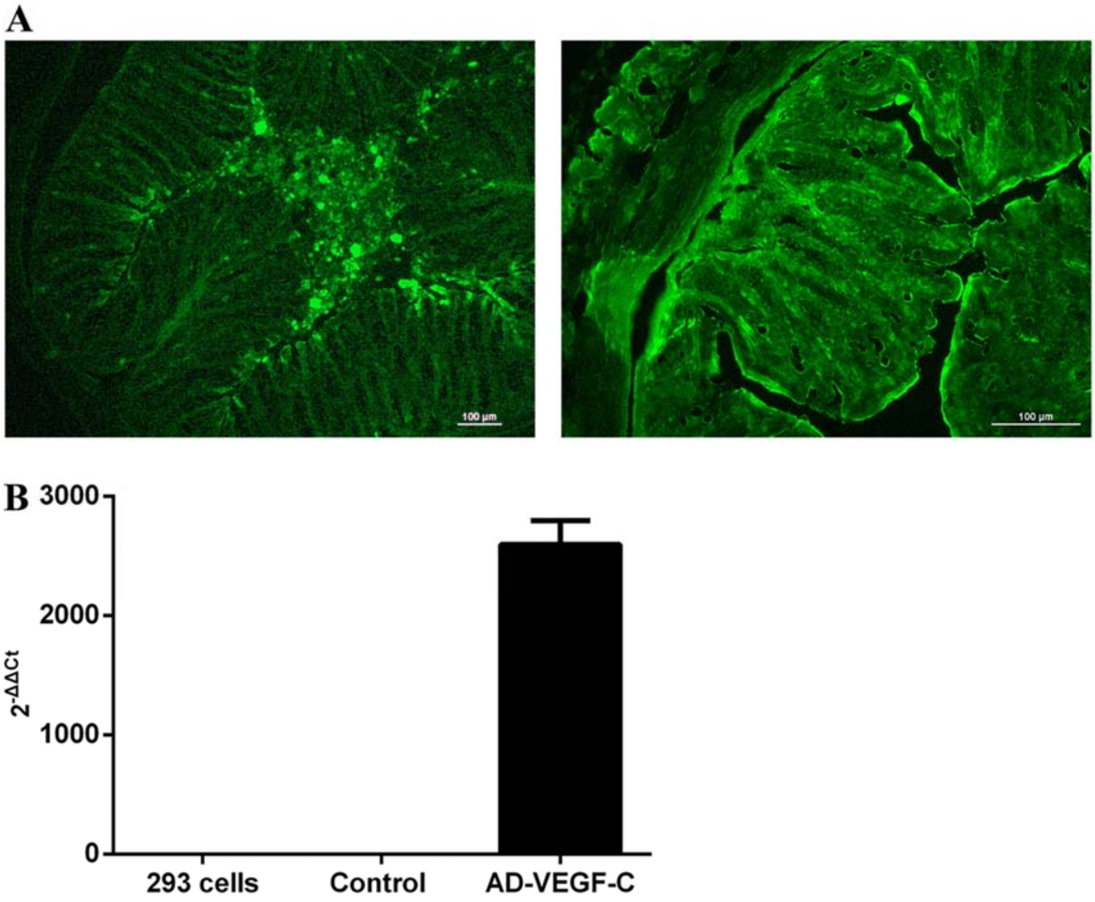

Figure S2. Representative fluorescence micrographs of dendritic cells in DSS-induced acute colitis. There was a significantly higher number of CD11c-positive dendritic cells in the AD-VEGF-C-treated mice (*A* - *a*, *b*, *B*; P=0.004) and recombinant VEGF-C156S-treated mice (*A* - *c*, *d*, *C*; P=0.002), compared to DSS-treated and PBS-treated mice, respectively. Data are reported as means±SD. *P<0.05 compared to DSS- or PBS-treated mice (n=5/group). †P<0.05 compared to water-treated mice (n=5/group). Statistical analysis was performed by ANOVA and Bonferroni's *post-hoc* test. AD-VEGF-C: adenovirus vascular endothelial growth factor-C; DSS: dextran sodium sulfate.

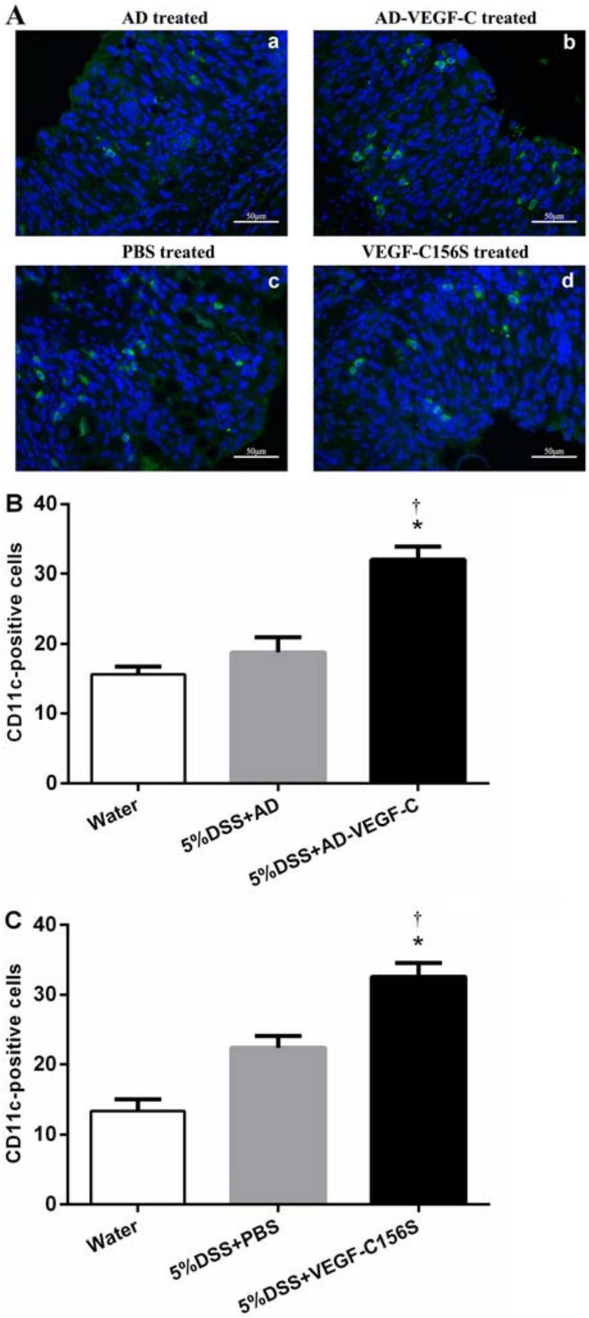

Figure S3. Representative fluorescence micrographs of neutrophils in DSS-induced acute colitis (*A*). Comparison of myeloperoxidase (MPO)-positive neutrophils between AD-VEGF-C-treated and DSS-treated mice (*B*) and between recombinant VEGF-C156S-treated and PBS-treated mice (*C*). There was no significant difference in the number of MPO-positive neutrophils in the colonic mucosa between the AD-VEGF-C-treated and control mice ($P>0.05$). However, VEGF-C156S-treated mice had a significantly higher number of MPO-positive neutrophils compared to PBS-treated mice ($P=0.018$). Data are reported as means±SD. $^{*}P<0.05$ compared to DSS- or PBS-treated mice (n=5/group). $^{\dagger}P<0.05$ compared to water-treated mice (n=5/group). Statistical analysis was performed by ANOVA and Bonferroni's *post-hoc* test. AD-VEGF-C: adenovirus vascular endothelial growth factor-C; DSS: dextran sodium sulfate.

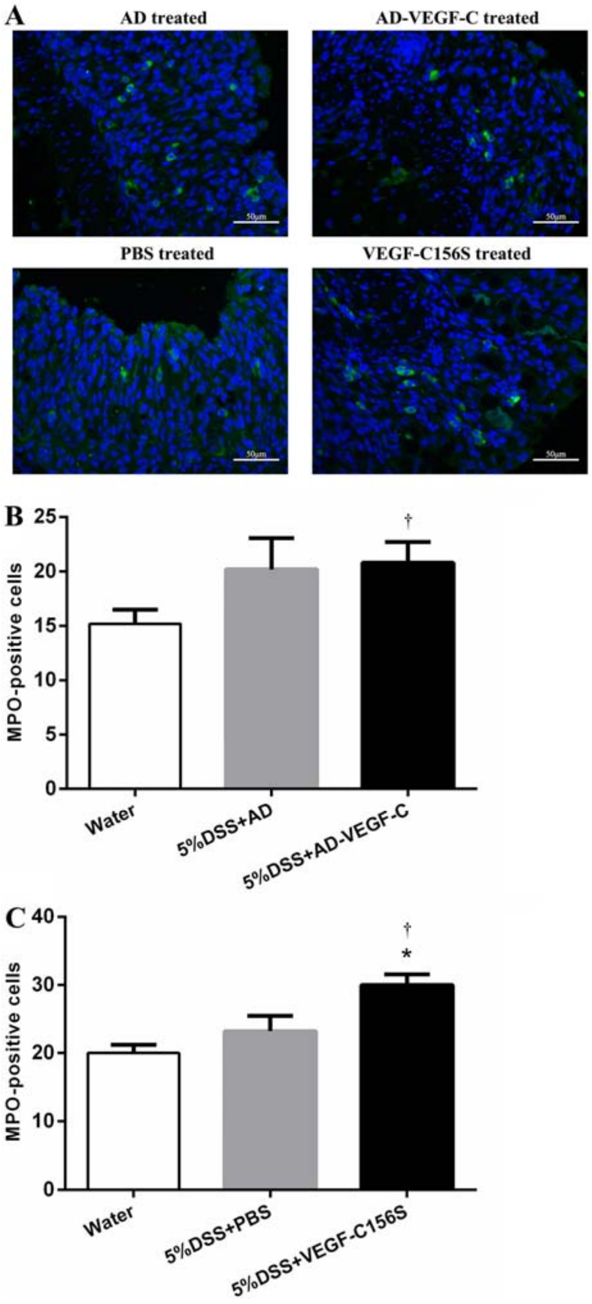

Figure S4. Comparison of colon thickness between AD-VEGF-C-treated and DSS-treated mice (*A*) and between AD-VEGF-C156S-treated and PBS-treated mice (*B*). Colon thickness was significantly increased in VEGF-C-treated and VEGF-C156S-treated mice compared to DSS- and PBS-treated mice, respectively (both P<0.001). Data are reported as means±SD (n=5/group). *P<0.05 compared to DSS- or PBS-treated mice. †P<0.05 compared to water-treated mice. Statistical analysis was performed by ANOVA and Bonferroni's *post-hoc* test. AD-VEGF-C: adenovirus vascular endothelial growth factor-C; DSS: dextran sodium sulfate.

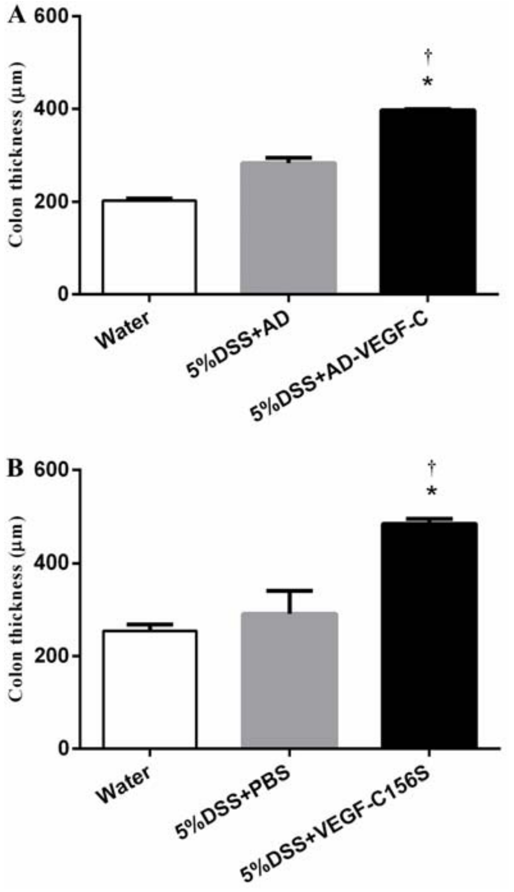

Supplement: Supplementary file 1 [file 1414-431X-bjmbr-1414-431X20154738-S1.pdf]
